# Different Alterations of Cerebral Regional Homogeneity in Early-Onset and Late-Onset Parkinson's Disease

**DOI:** 10.3389/fnagi.2016.00165

**Published:** 2016-07-12

**Authors:** Ke Sheng, Weidong Fang, Yingcheng Zhu, Guangying Shuai, Dezhi Zou, Meilan Su, Yu Han, Oumei Cheng

**Affiliations:** ^1^Department of Internal Medicine, The University Hospital, Chongqing UniversityChongqing, China; ^2^Department of Radiology, the First Affiliated Hospital, Chongqing Medical UniversityChongqing, China; ^3^Department of Neurology, The First Affiliated Hospital, Chongqing Medical UniversityChongqing, China

**Keywords:** early-onset Parkinson's disease, late-onset Parkinson's disease, regional homogeneity, putamen, resting-state functional MRI

## Abstract

**HIGHLIGHTS**

- Eighteen EOPD, 21 LOPD and 37 age-matched normal control subjects participated in the resting state fMRI scans.
- Age at onset of PD modulates the distribution of cerebral regional homogeneity during resting state.
- Disproportionate putamen alterations are more prominent in PD patients with a younger age of onset.

**Objective:** Early-onset Parkinson's disease (EOPD) is distinct from late-onset PD (LOPD) as it relates to the clinical profile and response to medication. The objective of current paper is to investigate whether characteristics of spontaneous brain activity in the resting state are associated with the age of disease onset.

**Methods:** We assessed the correlation between neural activity and age-at-onset in a sample of 39 PD patients (18 EOPD and 21 LOPD) and 37 age-matched normal control subjects. Regional homogeneity (ReHo) approaches were employed using ANOVA with two factors: PD and age.

**Results:** In the comparisons between LOPD and EOPD, EOPD revealed lower ReHo values in the right putamen and higher ReHo values in the left superior frontal gyrus. Compared with age-matched control subjects, EOPD exhibited lower ReHo values in the right putamen and higher ReHo values in the left inferior temporal gyrus; However, LOPD showed lower ReHo values in the right putamen and left insula. The ReHo values were negatively correlated with the UPDRS total scores in the right putamen in LOPD, but a correlation between the ReHo value and UPDRS score was not detected in EOPD.

**Conclusions:** Our findings support the notion that age at onset is associated with the distribution of cerebral regional homogeneity in the resting state and suggest that disproportionate putamen alterations are more prominent in patients with a younger age of onset.

## Introduction

Early-onset Parkinson's disease (EOPD) usually refers to the form of PD in which the first symptoms appear before 40 years of age, but some studies consider a disease onset of up to 50 years of age to be EOPD (Schrag and Schott, 2006). The prevalence of early-onset PD is approximately 5–10% of the entire PD patient population (Quinn et al., 1987). EOPD had been long contrasted with the late-onset form of the disease (LOPD) until they became united under the same eponym (Spica et al., 2013). Although these two forms of PD now refer to a single disease, previous studies have reported clinical and neuroimaging differences between EOPD and LOPD. EOPD tends to have slower disease progression than LOPD, particularly with regard to falls and freezing (Inzelberg et al., 2004; Alves et al., 2005). Schrag et al. have noted that EOPD is associated with less cognitive decline (Schrag et al., 1998), at least until patients reach a more advanced age, and EOPD patients experience poorer social adjustment and higher rates of depression and anxiety (Schrag and Schott, 2006; Mehanna et al., 2014). In contrast, more patients with EOPD have earlier motor complications, such as dyskinesias, dystonia, and motor fluctuations (Schrag et al., 1998). With respect to treatment, EOPD patients have a better medication response to levodopa (Ribeiro et al., 2009). Moreover, Kondo et al. have suggested that the dopamine agonist therapies that are more commonly used in EOPD might reduce dopamine transporter (DAT) loss during PD disease progression (Kondo, 2002). The pathophysiological mechanisms that differentiate EOPD and LOPD disease progression are not yet clear (Shih et al., 2007).

Little is known about how these two clinical subtypes differ at the neural level. The results of structural imaging in EOPD are typically normal (Schrag and Schott, 2006). Functional imaging with single-photon emission computed tomography (SPECT) in EOPD reveals similar findings to that in classic PD with presynaptic dysfunction of nigrostriatal dopaminergic neurons (Thobois et al., 2003). However, positron emission tomography (PET) indicates that the age of disease onset might be one of the factors related to the heterogeneity in patterns of regional metabolism (Shih et al., 2007; de la Fuente-Fernández et al., 2011). Compensatory up-regulatory functions of postsynaptic receptors may modify disease severity and the degrees of the main clinical symptoms of EOPD observed by PET (Nagasawa et al., 1996). An MRI study of brain iron has highlighted that there is dysregulation of iron metabolism in the substantia nigra that differs in early- vs. late-onset PD (Bartzokis et al., 1999). Taken together, these findings suggest that the topographic distributions of pathological changes differ in EOPD and LOPD. However, the available literature on this topic lacks clarity because adjustments for multiple types of testing are insufficient, direct comparisons between EOPD and LOPD have not been performed, an a priori hypothesis has not been established, and different image analysis techniques have been used.

In the present study, we used regional homogeneity (ReHo) to detect different patterns of abnormal neural activity in early- and late-onset PD patients compared with age-matched normal controls. We hypothesized that a different level of regional brain dysfunction would be detected in the EOPD group compared to the LOPD group, even at similar disease stages.

## Materials and methods

We studied 39 patients with PD (16 males, 23 females; mean age 59.5) and 37 healthy, age- and gender-matched controls (mean age 59.7). All patients met the following criteria: (1) All fulfilled the UK Parkinson's Disease Society Brain Bank criteria for idiopathic PD; (2) The age of onset was either before or after 50 years [a commonly used cut-off age point to divide EOPD from LOPD (Schrag and Schott, 2006)]; (3) a H&Y stage equal to or less than 3.0 while in an “off” state; (4) The Mini-Mental State Examination (MMSE) scores were higher than 24; (5) The was no evidence of primary medical illness, psychiatric illness or neurological illness; (6) The T2-weighted MRI images did not exhibit gross white matter or gray matter alterations; (7) To exclude the confounding factor of a long illness duration and motor complications, the illness duration was less than 10 years and without motor complications; (8) To avoid disturbances to the fMRI signal, all patients had, at most, a mild tremor.

Neurological and psychiatric evaluations were conducted during the “off” medication state (wherein subjects refrained from taking their PD medications for at least 12 h prior to assessment) and included the Hoehn and Yahr (H&Y) scale, the unified Parkinson's disease rating total scale (UPDRS), the UPDRS Part III, the MMSE, and the HAMD. All neuropsychological evaluations and RS-fMRI scans (for ReHo and functional connectivity analyses) were implemented around the same time.

Thirty-seven age- and gender-matched normal controls were recruited. They were either younger than 50 years (19 persons) or older than 50 years (18 persons). All normal control subjects had a normal neurological status and were without a history of serious medical or neuropsychiatric disease or a family history of psychiatric or neurological disease among first-degree relatives.

Our research protocol complied with the guidelines for the conduct of research involving human subjects as established by the National Institutes of Health and the Committee on Human Research at Chongqing Medical University in China.

### Data acquisition

All MR images were acquired using a GE Signa HDxt 3.0T scanner (General Electric Medical Systems, USA) with a standard 8-channel head coil. Foam padding was used to minimize head motion. Apart from this, no other special methods were employed to prevent head movement. During RS-fMRI acquisition, all subjects were instructed to relax and to keep still with eyes closed but to remain awake (confirmed with post-scan debriefing).

RS-fMRI data were acquired using an echo-planar image (EPI) pulse sequence and the following parameters: 33 axial slices; thickness/gap = 4.0/0 mm; matrix = 64 × 64; TR = 2000 ms; TE = 40 ms; flip angle = 90°; and FOV = 240 × 240 mm. A total of 240 time points were obtained in 8 min. High-resolution 3D-T1 images were also acquired (repetition time [TR] = 8.3 ms; echo time [TE] = 3.3 ms; flip angle = 15°; thickness/gap = 1.0/0 mm; field of view [FOV] = 240 × 240 mm; matrix = 256 × 192).

### Data processing

The data were analyzed using Statistical Parametric Mapping (SPM8) (http://www.fil.ion.ucl.ac.uk), Resting State fMRI Data Analysis Toolkit (REST) software on version 1.8 (Song et al., 2011) (http://www.restfmri.net), and the Data Processing Assistant for Resting-State fMRI–Advanced on version 2.1 (DPARSFA; http://www.restfmri.net) with Matlab version 7.10.0.499 (Machizawa et al., 2010).

The first 10 time points were discarded to account for scanner calibration and the acclimatization of subjects to the scanning environment, after which, 230 time points remained. The following preprocessing procedures were included: Time alignment across slices, motion correction, within-subject registration between T1 and EPI images, T1 segmentation, and the application of normalization parameters to the BOLD fMRI datasets to register them to Montreal Neurologic Institute (MNI) space, with voxels resampled at 3 × 3 × 3 mm. Linear trends were removed, and a temporal filter (0.01 < *f* < 0.08 Hz) was applied to eliminate low-frequency drift and physiological high-frequency noise. The high-resolution 3D-T1 images were segmented into gray matter (GM), whiter matter (WM), and cerebrospinal fluid (CSF) by using unified segmentation (Ashburner and Friston, 2005) and were then normalized to MNI space. Head motion can influence on result even though traditional realignment was performed (Power et al., 2012; Satterthwaite et al., 2013). All images were realigned to the first image to account for head motion. All subject had a maximum displacement in any of the cardinal directions (*x, y, z*) less than 2 mm, or a maximum spin (*x, y, z*) less than 2°. In addition, following previous studies (Van Dijk et al., 2012), the mean relative displacement was used to measure subjects' head motion in scanner.

### ReHo analysis

Individual ReHo maps were generated for each subject using REST software; Kendall's coefficient of concordance (KCC) was calculated at each voxel to establish similarities between the time series of each specific voxel and its 26 neighboring voxels within a whole-brain mask (this mask is provided by DPARSFA, excluding non-brain areas). The KCC value was calculated to this voxel, and an individual KCC map was obtained for each subject. To reduce the influence of individual variations in the KCC value, ReHo map normalizations were performed by dividing the KCC among each voxel by the averaged KCC of the whole brain (using the same whole brain mask). The calibrated ReHo maps were further smoothed using an isotropic Gaussian kernel with a full-width at half maximum (FWNM) of 4 × 4 × 4 mm.

### Statistical analysis

Differences of age and MMSE scores among the four groups were compared by using analysis of variance (ANOVA) with two factors: Parkinson's disease and age of onset. The Pearson χ^2^-test was applied to compare gender differences. Student's *t*-test was employed to compare illness duration, UPDRS scores and H&Y stages between the two patient groups.

To identify the ReHo maps between groups, a full factorial design was used that automatically models interactions between the factors in SPM8. We used diagnosis and age as factors with independent levels with unequal variance. To test group differences *post-hoc*, we used separate 2-sample *t*-tests to compare early-onset PD patients with young control subjects, and late-onset PD patients with old control subjects. Finally, the threshold for statistical significance in ReHo *post-hoc* analysis was set to *p* < 0.01 with family wise error (FWE) correction at the voxel level.

### Correlation between clinical data and ReHo

To examine the association of ReHo abnormalities with the clinical data of the PD patients, we also performed a voxel-based correlative analysis between mean ReHo values extracted from the regions with significant group differences of the clusters (EOPD vs. LOPD), patient illness durations and total UPDRS scores. For correlation analysis we used an uncorrected threshold of *p* < 0.001.

## Results

### Demographic and clinical data

Demographic information, illness duration, disease stage, UPDRS scores, and medications are summarized in Table 1 for all patients (18 EOPD and 21 LOPD) and control subjects (19 young NCs, and 18 old NCs). There was a significant difference in age among the four groups (ANOVA, *P* < 0.01). The young and old NCs were matched with EOPD and LOPD patients in age (*t*-tests EOPD *P* = 0.39; LOPD *P* = 0.21) and gender (Chi-square test EOPD *P* = 0.62; LOPD *P* = 0.50). There was no difference between the EOPD and LOPD group in terms of the duration of PD, the UPDRS total score, the UPDRS Part III score, the side initially affected, the medication used, or the MMSE score (two-sample *t*-tests, *P* > 0.05).

**Table 1:** **Demographic and clinical data in EOPD/LOPD patients and normal controls**.

**Group N**	**EOPD**	**Young NC**	**EOPD vs. controls *P*-value**	**LOPD**	**Old NC**	**LOPD vs. controls *P*-value**	**EOPD vs. LOPD *P*-value**
N (female)	18 (8)	19 (10)	0.62♦	21 (9)	18 (10)	0.50♦	0.92♦
Age (Y)	45.4 ± 6.07	45.8 ± 3.55	0.39▲	63.6 ± 4.84	61.7 ± 9.73	0.21▲	<0.001⋆
Disease duration (Y)	3.04 ± 1.99	NA		3.1 ± 1.78	NA		0.43▲
Disease stage(H&Y)	2.03 ± 0.78	NA		2.0 ± 0.62	NA		0.49▲
UPDRS	37.8 ± 9.86	NA		39.3 ± 10.62	NA		0.3▲
UPDRS III	16.94 ± 5.07	NA		18.61 ± 4.51	NA		0.29▲
MMSE	27.8 ± 1.56	29.2 ± 0.91	0.003▲	26.5 ± 2.16	27.2 ± 2.74	0.21▲	0.065⋆
HAMD	6.38 ± 2.06	5.47 ± 1.87	0.089▲	6.52 ± 2.81	5.72 ± 2.10	0.17▲	<0.001⋆
Side initially affected, R/L	11/7	NA		10/11	NA		0.40●
L-Dopa dose (mg/d)	395.8 ± 278.7	NA		440.5 ± 187.9	NA		0.28▲
No. (%) of patients treated with pramipexole	14 (78)	NA		16 (76)	NA		0.91●
No. (%) of patients treated with piribedil	9 (50)	NA		14 (67)	NA		0.29●

*NC, Normal control; EOPD, Early-onset Parkinson's disease patients; LOPD, Late-onset Parkinson's disease patients*.

*▲: Two sample t-test*.

*●: Pearson χ^2^-test*.

*⋆: ANOVA analysis with two factors*.

*♦: Chi-square test*.

### Head motion

There was no significant difference in head motion when the mean head motion was measured and compared between the four groups using ANOVA (*P* = 0.21). Therefore, PD patients and normal controls were similar in head motion characteristics.

### ReHo

The full factorial design showed that diagnosis and age affected the ReHo value patterns. A diagnosis of PD was associated with an alternative ReHo in the bilateral gyrus rectus, left caudate nucleus, bilateral putamen, bilateral inferior frontal gyrus, and bilateral cerebellum (*p* < 0.05, FWE-corrected). Older age was associated with an alternative ReHo in the bilateral superior frontal gyrus orbital part (*p* < 0.05, corrected by FWE). Furthermore, there was a significant interaction between age and diagnosis (*p* < 0.05, FWE-corrected), showing that with a younger onset of PD, there was an altered brain ReHo value in the left superior frontal gyrus, right putamen, right superior frontal gyrus, left inferior temporal gyrus, right fusiform, and left insula when compared to that in late-onset PD patients.

**Figure 1:**
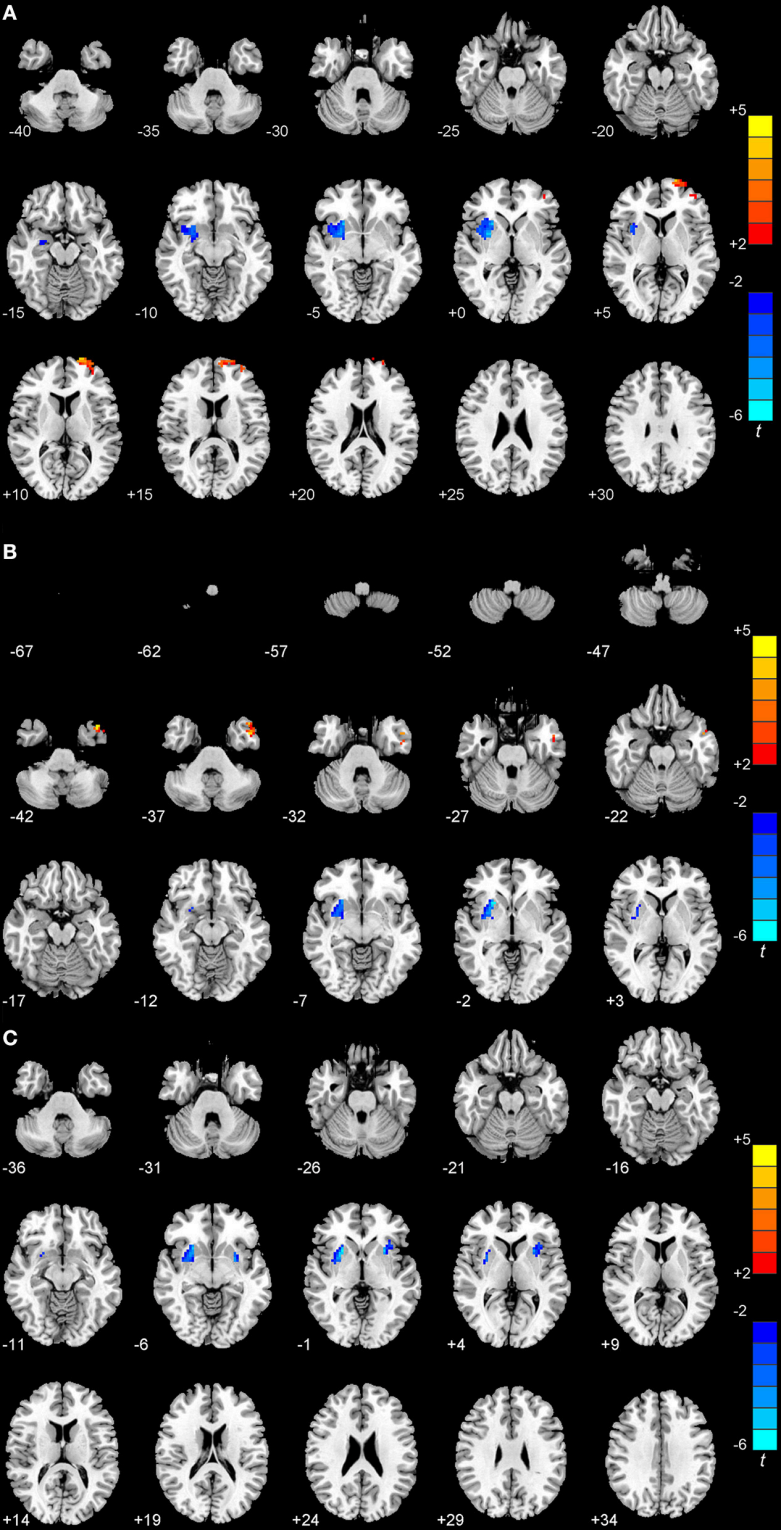
**Statistical maps showing the ReHo differences among patients with EOPD or LOPD and normal controls (***P*** < 0.01, FWE corrected). (A)** Compared with LOPD, EOPD showed significantly deceased ReHo values in the right putamen and increased ReHo values in the left superior frontal gyrus. **(B)** Compared with younger normal controls, EOPD showed significantly deceased ReHo values in the right putamen and increased in the left inferior temporal gyrus. **(C)** Compared with older normal controls, LOPD exhibited decreased ReHo values in the right putamen and left insula. Red and blue denote higher and lower ReHo values, respectively, and the color bars indicate the *T*-value from *post-hoc* analyses between each pair of groups.

In the *post-hoc* comparison between early-onset and late-onset PD patients, we observed that EOPD patients showed a significant decrease in the ReHo value in the right putamen and an increase in the ReHo value in the left superior frontal gyrus (*p* < 0.01, FWE-corrected; Figure 1A). The ReHo value in EOPD patients was lower than that in age-matched controls in the right putamen and higher in left inferior temporal gyrus (*p* < 0.01, FWE-corrected; Figure 1B). Compared to older normal controls, patients with LOPD exhibited decreased ReHo values in the right putamen and left insula (*P* < 0.01; FWE-corrected; Figure 1C and Table 2).

**Table 2:** **Regions showing ReHo differences among the EOPD, LOPD, and NC groups by ***post-hoc*** analysis**.

**Brain region**	**Cluster size**	**MNI**			***T*-value**
		***x***	***y***	***z***	
**EOPD** < **LOPD**					
Right putamen	99	29	14	0	−4.23
**EOPD** > **LOPD**					
Left superior frontal gyrus	106	−25	65	10	5.57
**EOPD** < **YOUNG NC**					
Right putamen	93	28	7	−3	−7.00
**EOPD** > **YOUNG NC**					
Left inferior temporal gyrus	139	−44	10	−39	5.67
**LOPD** < **OLD NC**					
Right putamen	95	24	9	−5	−5.40
Left insula	112	−33	21	−1	−5.49

### Correlations between ReHo values and the clinical data

**Figure 2:**
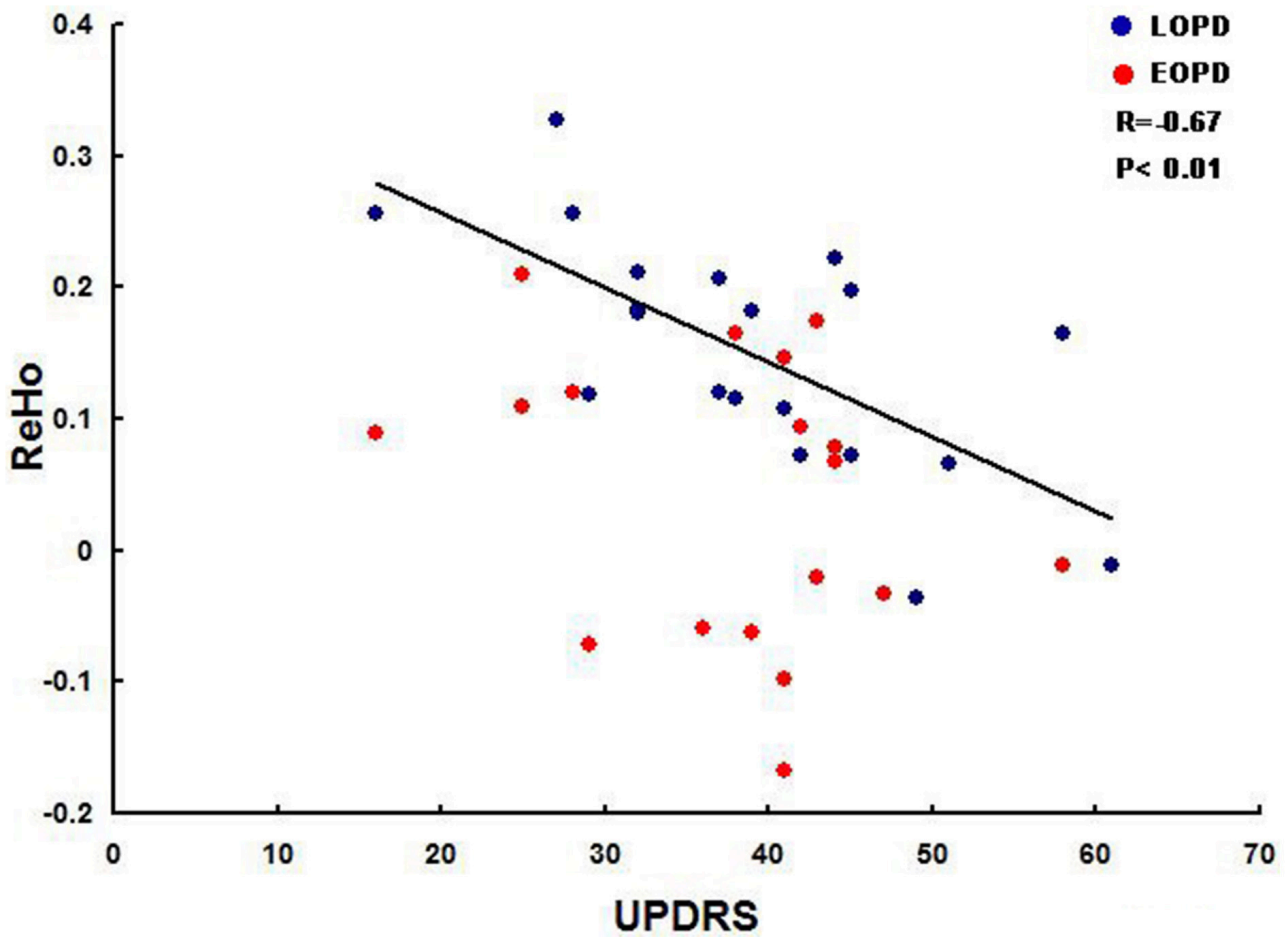
**Correlation between the mean fitted ReHo index and the UPDRS score in both EOPD and LOPD patients (***P*** < 0.01, uncorrected)**. A negative correlation was discovered between the ReHo values of the right putamen (*x, y, z* = 29, 14, 0) and the total UPDRS scores in LOPD patients (*r* = –0.67, *p* < 0.01).

A negative correlation was found between the ReHo values of the right putamen (*x, y, z* = 29, 14, 0) and the UPDRS scores in LOPD patients (*r* = –0.67, *p* < 0.01). In LOPD patients, no significant correlation was discovered between ReHo values and illness duration. In EOPD patients, no significant correlation was observed between the ReHo value and UPDRS total scores. Neither was there a correlation between the ReHo value and disease duration in the EOPD group (*P* < 0.01, uncorrected; Figure 2).

## Discussion

The present fMRI study used the ReHo method to investigate the regional activity alterations in EOPD and LOPD patients compared with age-matched normal controls in the resting state. We found different regional activity patterns between EOPD and LOPD patients. Compared with age-matched normal controls, EOPD, and LOPD patients showed altered activities mainly in the basal ganglia. Furthermore, compared to LOPD patients, EOPD patients exhibited decreased ReHo values in the right putamen and increased ReHo values in the left superior frontal gyrus despite having similar disease severity. Additionally, using voxel-wise correlation analysis, in LOPD patients, we found a negative correlation between the ReHo value in the right putamen and the UPDRS total score, but this was not detected in EOPD patients.

In previous studies, RS-fMRI has detected spontaneous neuronal activity (Biswal et al., 1995) and/or the endogenous or background neurophysiologic processes of the brain based on the temporal correlation of spontaneous, blood oxygen level-dependent (BOLD) fluctuations in low frequencies (< 0.08 Hz; Raichle et al., 2001; Fox and Raichle, 2007). ReHo is a RS-fMRI analysis method based on a data-driven approach; therefore, it requires no prior knowledge and has good test-retest reliability (Zuo et al., 2013). ReHo measures the functional coherence of a given voxel with its nearest neighbors and evaluate resting-state brain activities based on the hypothesis that significant brain activities would more likely occur in clusters than in a single voxel (Zang et al., 2004). Decreased or increased ReHo suggests that functional activity in certain regions is poorly or highly synchronized compared to controls (Su et al., 2015). ReHo measurement of RS-fMRI has been employed by others to analyze the similarities of intra-regional time series across the whole brain of the regional BOLD signal. Few studies have used whole-brain functional approaches such as regional homogeneity (ReHo) to investigate the functional differences between EOPD and LOPD.

The putamen, which has shown decreased ReHo in both types of PD patients, is one of the important regions associated with planning and execution of self-initiated movements (François-Brosseau et al., 2009). Previous studies have demonstrated that the putamen had lower cerebral blood flow and was hypoactivated in PD when various movements were performed compared to normal subjects (Playford et al., 1992; Rowe et al., 2002). Compared to normal controls, a significant reduction of presynaptic dopaminergic markers in the putamen have been observed in previous reports of EOPD patients (Ribeiro et al., 2009). In our study comparing ReHo maps between two PD groups and young or old NCs, we also observed PD-related pathophysiology and found altered activities in PD that were mainly focused in the putamen. Our finding of less functional synchronization of the putamen in EOPD and LOPD patients is consistent with a previous study on PD using the ReHo method (Wu et al., 2009a).

EOPD cases had lower dopamine transporter (DAT) binding in the putamen than did LOPD cases with similar disease duration (Antonini et al., 2002). In previous studies, although EOPD patients have shown a lower DAT density, they have presented similar symptoms of motor disability and PD severity during the off state (Shih et al., 2007). Consistent with these studies, we found that no significant difference in disease severity or disease duration exists between the two PD groups; however, EOPD patients showed decreased ReHo values in the right putamen compared to LOPD patients. These observations suggest that EOPD patients may have more efficient compensatory mechanisms, which is in alignment with previous studies (Pavese et al., 2009; de la Fuente-Fernández et al., 2011). Compensatory mechanisms in the dopaminergic and/or non-dopaminergic systems, pharmacokinetic adaptations, and neuronal plasticity might be more effective in EOPD patients (Shih et al., 2007). The increase in dopamine neuronal loss and the early neuroplastic compensatory mechanisms might explain why EOPD patients have less functional synchronization in the putamen compared to LOPD patients despite similar symptoms and disease stage. This might be one of the reasons for the differences shown in several clinical reports (Alves et al., 2005; Ribeiro et al., 2009). Patients with EOPD tend to exhibit slower disease progression and respond better to levodopa medications compared to LOPD patients. Furthermore, we found a significant negative correlation between the UPDRS total score and ReHo value within the putamen in LOPD patients that was not discovered in EOPD patients. Consistent with our results, Wu et al. have shown that an altered ReHo value in the putamen correlates with the progress of the disease in elderly PD patients (Wu et al., 2009a). A PET study has also observed that a significant negative correlation between DAT in the putamen and clinical stage, as determined by H&Y staging, was more markedly observed in an LOPD group compared to an EOPD group (Nagasawa et al., 1996). We found that the severity of PD, as measured by the UPDRS total score (and UPDRS Part III score), was not significantly different between the two patient groups, although EOPD appeared to have lower spontaneous activity. This finding is in agreement with those of other studies. Our results increase the evidence to support that PD-related changes occur in the putamen and disproportionate putamen alterations are more prominent in patients with a younger age of onset. However, whether ReHo could be a predictor of disease progression and an available marker for underlying disease is unclear, and needs further investigation.

We found increased ReHo values in the left superior frontal gyrus in EOPD patients compared to patients with LOPD. The superior frontal gyrus is thought to be related to feelings of hate (Zeki and Romaya, 2008) and emotion control. There are several possible explanations that might underlie our findings. First, the hyperactivation of this region might be a compensatory mechanism in EOPD. The altered volume and region activity in this area may be characteristics changes in patients with early PD (Long et al., 2012; Yang et al., 2013). The pattern of hyperactivation in prefrontal cortex in patients with early PD has been shown to drop progressively over time, and it might correlate with the progression of the disease (Baglio et al., 2011). When considered together with our results, hyperactivity in the superior frontal gyrus might indicate a functional compensation for a defective basal ganglia. Second, the different patterns of spontaneous brain activity might reflect a difference in regional vulnerability across the PD subgroups. The frontal gyrus is involved in both control of motor processing (Tanei et al., 2009) and attention to action (Jueptner et al., 1997; Rowe et al., 2002). The associative circuit in the striatal-thalamo-cortical loop projects from associative cortical areas, such as the prefrontal cortex, to the caudate nucleus, and the putamen (Baglio et al., 2011). It is generally accepted that inhibition of the motor response is principally mediated by the fronto-striatal-thalamic pathway. The abnormalities of the superior frontal cortex indicate a selective impairment of the fronto-striatal-thalamic loop, which is probably due to a reduced level of dopamine in the basal ganglia (Mink, 1996) or frontal lobes that are secondary to abnormal basal ganglia outflow. Additionally, the frontal gyrus appears to be important in cognition and emotion regulation (Jueptner et al., 1997; Rowe et al., 2002; Phillips et al., 2003). A previous resting state functional imaging study has demonstrated that PD is characterized by increased regional spontaneous neural activity in the frontal area (Luo et al., 2014). Further, previous clinical investigations have observed that EOPD patients have higher rates of mood disorder (Schrag and Schott, 2006). An earlier study by our group on PD accompanied by depressive symptom (PDD) have observed significantly increased regional activity in frontal gyrus in PDD compared with the non-depressed PD patients (Sheng et al., 2014). When combined with our results, despite we have excluded the patients with cognitive and emotional disorders in our study, these findings imply that altered neural activity in the frontal cortex might be associate with higher rates of depression in EOPD.

Herein, we also observed that compared with age-matched control subjects, EOPD patients showed increased ReHo values in the left inferior temporal gyrus. The dysfunction of the temporal cortex underlies PD patient difficulty in dealing with complex sentence structures (Ye et al., 2012). In untreated PD patients, the earliest pathophysiological changes have been shown to occur in the subcortical white matter of the temporal gyrus (Martin et al., 2009). However, while previous studies have found changes in structure and function in the temporal gyrus area, age has not been considered as a factor in previous studies. Thus, more conclusive studies are still needed. Two VBM studies have observed that temporal lobe atrophy occurs in early-onset depressive disorders (Greenwald et al., 1997) and in PD patients with depression (Phillips et al., 2003). When combined with another clinical study (Mehanna et al., 2014), depression is more frequent in EOPD patients; therefore the higher ReHo values in EOPD patients suggest that altered temporal gyrus activity might be associated with emotion regulation in EOPD. However, additional studies are needed to confirm these conclusions.

We also observed that the LOPD group showed significantly lower ReHo values in the left insula compared with age-matched control subjects. Previous neuroimaging studies using structural MRI, fMRI, or PET have observed insula damage in PD patients, and these patients also exhibited fatigue or dysarthria (Kikuchi et al., 2001; Stefurak et al., 2003; Pavese et al., 2010). A functional connectivity study on PD patients has found some regions that showed positive functional connectivity with the insula, including the putamen and supplementary motor area (Yu et al., 2013). These results provide a promising aspect to investigate the pathophysiological characteristics of LOPD. More studies need to be conducted to reveal the initial state of ReHo in the insula and its relevance with respect to age in PD.

Several limitations of the present study deserve to be mentioned. First, the number of experimental subjects was relatively small. The small sample size may reduce the generalizability of our results. Replication of the results with larger samples will be necessary to establish the reliability of the differences between the EOPD group and LOPD group. A larger independent data set will be required to validate the current findings. Second, it has been shown that L-DOPA might influence brain activity over time (Wu et al., 2009b; D'Andrea et al., 2013). In our study, all patients with PD were assessed after stopping their medication for 12 h prior to scanning to minimize the impact of medicine. However, the potentially confounding effects of the chronic use of dopaminergic medications could not be avoided herein because an absolute elimination of the influence of medications was impossible. Additionally, it is known that head motion can influence results even when traditional realignment is performed (Power et al., 2012; Satterthwaite et al., 2013). In the current study, all images were realigned to the first image to account for head motion. Any subject was excluded who had a maximum displacement in any of the cardinal directions (*x, y, z*) that was larger than 2 mm or a maximum spin (*x, y, z*) that was larger than 2°. Finally, we noted that the abnormal activity that was observed in some brain regions of EOPD and LOPD subjects only demonstrated regional synchronization changes that were associated with neural spontaneous activity. However, the functional connectivity and global complex brain network properties of resting state neural activity remain to be addressed.

Taken together, our findings support the notion that age at onset modulates the distribution of cerebral regional homogeneity in resting state and that disproportionate putamen alterations are more prominent in PD patients with a younger age of onset.

## Author contributions

Conceived and designed the experiments: OC, KS, and WF. Performed the experiments: KS, WF, YZ, and GS. Analyzed the data: KS, WF, YZ, and GS. Contributedreagents/materials/analysis tools: WF, YH, and DZ. Wrote the paper: KS, WF, and OC. The author denies that he has any intention to obtain any financial interests.

### Conflict of interest statement

The authors declare that the research was conducted in the absence of any commercial or financial relationships that could be construed as a potential conflict of interest.
